# Women with familial risk for breast cancer have an increased frequency of aldehyde dehydrogenase expressing cells in breast ductules

**DOI:** 10.1186/1472-6890-13-28

**Published:** 2013-11-04

**Authors:** Björn L Isfoss, Bo Holmqvist, Helena Jernström, Per Alm, Håkan Olsson

**Affiliations:** 1 Department of Oncology, Clinical Sciences Lund, Lund University, SE-221 85 Lund, Sweden; 2 Department of Pathology, Telemark Hospital, 3710 Skien, Norway; 3 Department of Pathology, Skane University Hospital, SE-221 85 Lund, Sweden; 4 ImaGene-iT AB, Medicon Village, SE-223 81 Lund, Sweden; 5 Department of Pathology, Clinical Sciences Lund, Lund University, SE-221 85 Lund, Sweden; 6 Department of Cancer Epidemiology, Clinical Sciences Lund, Lund University, SE-221 85 Lund, Sweden

**Keywords:** Stem cells, Aldehyde dehydrogenase, Breast neoplasia, Familial cancer, *BRCA1*, *BRCA2*, Histology, Immunohistochemistry, Breast ductules

## Abstract

**Background:**

Knowledge is limited regarding the association between stem cells in histologically benign breast tissue and risk factors for breast cancer, and hence we addressed this issue in the present study. Recently, we assessed the histology of benign breast tissue from cancer and non-cancer patients for cells positive for the putative stem cell marker aldehyde dehydrogenase 1 A1 (ALDH), and the findings indicated an association between expression of ALDH and the hormonal factors menopause and hormone therapy. The current investigation examined possible associations between various known clinical and genetic risk factors for breast cancer and cellular expression of ALDH in ductules in benign human breast tissue.

**Methods:**

The study included breast surgery patients that were *BRCA1/2* mutation carriers without breast cancer (*n* = 23), had *BRCA1/2* (*n* = 28) or sporadic (*n* = 21) breast cancer, or required non-cancer-related mammoplasty (*n* = 34). The distribution and frequency of ALDH-immunolabelled cells were correlated to patient subgroups with different risk factors, using mammoplasty patients as a control group. Statistical analyses comprised linear and logistic regression, Spearman’s rank test, Pearson’s test, and Fisher’s exact test. In two-tailed tests, *p* < 0.05 was considered significant.

**Results:**

A strong association was found between family history of breast cancer and a high frequency of ALDH+ cells (*p* = 0.001) at all ductular levels in all groups, regardless of *BRCA* status, age, parity, or occurrence of cancer. In pre-menopausal non-*BRCA* cancer patients, the frequency of ALDH+ cells increased with age (*p* < 0.01) but decreased with increasing parity (*p* < 0.03). High frequencies of ALDH+ cells were found in the non-basal ductular levels in *BRCA1* mutation carriers (*p* = 0.03), but in the basal ductular level in *BRCA2* cancer patients (*p* = 0.02). Among post-menopausal patients, only on-going hormone replacement therapy was correlated with a high number of ALDH+ cells (*p* < 0.03).

**Conclusion:**

In histologically normal breast tissue, we found a positive association between the frequency of ductular ALDH+ cells and several breast cancer risk factors, particularly family history of this disease, which supports previous evidence that ALDH plays a role in breast cancer.

## Background

A stem-like cancer cell is defined as a cell with the capacity for self-renewal and the ability to generate different cell types that form a tumor [1]. In 2003, the presence of benign stem cells in non-histological breast tissue preparations was identified on the basis of functional cell characteristics [2,3], and a brief histological description of these cells was published in 2007 [4]. It has been proposed that aldehyde-dehydrogenase-expressing (ALDH+) breast cells include malignant stem-like cell populations that maintain and cause progression of cancer [4,5]. ALDH catalyzes oxidation and is necessary for retinoic acid synthesis, and it has been suggested that this enzyme is also involved in stem cell preservation and initiation of differentiation [6]. Normal mammary stem cells and stem-like cancer cells were recently shown to express an alternative isoform of ALDH [7], but the stem cell properties or cancer-related functions of cells that are positive for any isoform still remain unclear.

Several clinical and molecular factors predict the risk of developing breast cancer. For example, the presence of *BRCA1* and *BRCA2* gene mutations confers a 40–80% lifetime risk [8], and exogenous hormone exposure is associated with relative risks of 1.5 to 2.0 [9]. Furthermore, non-malignant histological changes such as atypical ductal hyperplasia are associated with a 28% risk of breast cancer [10].

ALDH+ cells are found in up to 48% of breast cancer tumors and are believed to cause late recurrence, and these cells are also associated with an adverse prognosis and poor outcome after conventional anti-cancer drug treatment [5,11,12].

To assess the potential of ALDH as a predictive marker for subsequent development of breast cancer, it is necessary to define the normal ranges of frequencies and distribution of ALDH+ cells in histologically benign breast tissue in women with and without breast cancer. Such data are limited at present, although a small case-control study showed elevated levels of ALDH+ cells in epithelium and stroma of patients who later developed cancer [13], and an investigation of African women revealed a higher frequency of ALDH+ cells in breast cancer tissue compared to benign breast tissue [14]. It is also essential to determine whether the frequency or distribution of ALDH+ putative stem cells in histologically normal breast tissue is related to risk factors for breast cancer. We recently described in detail the distribution of ALDH+ cells in terminal duct lobular units (TDLUs) and stroma in benign breast tissues [15]. The correlations with risk factors that were observed in the small group of patients assessed in that investigation suggested that density and distribution of ALDH+ cells are associated with menopausal state and hormone replacement therapy. Therefore, our aim in the present study was to examine ALDH expression in ductular cells in a larger patient group and to elucidate the relationship between such expression and various risk factors for breast cancer, including *BRCA1/2* mutation status, familial breast cancer history, hormone intake, parity, and age at menarche.

## Methods

### Patient material

The patients considered for inclusion in this study were female patients who had been treated with breast surgery in Skåne County and met the criteria for one of the following groups:

Group A (*n* = 30): breast cancer patients without *BRCA1/2* mutation; surgery during the period 1999–2006.

Group B (*n* = 19): breast cancer patients with *BRCA1* mutation; surgery during the period 1984–2009.

Group C (*n* = 16): breast cancer patients with *BRCA2* mutation; surgery during the period 1984–2009.

Group D (*n* = 13): *BRCA1* mutation carriers without breast cancer; prophylactic mastectomy during the period 1996–2010.

Group E (*n* = 13): *BRCA2* mutation carriers without breast cancer; prophylactic mastectomy during the period 1996–2010.

Group F (*n* = 35): patients without breast cancer or *BRCA1/2* mutations; mammoplasty during the period 1993–1994.

Thus a total of 126 patients were reviewed for inclusion in our study. An experienced histopathologist (BLI) examined the original hematoxylin and eosin (H&E) stained microscope slides from each patient without knowledge of the clinical parameters. The tissue blocks containing the largest number of histologically normal TDLUs for each patient were selected for the investigation. Exclusion criteria were any of the following: patient received neoadjuvant therapy; no tissue blocks available in the archives; no tissue block contained ≥10 morphologically benign TDLUs. Based on the mentioned criteria, a total of 106 patients were included in the study (see Table 1).

**Table 1:** Patient groups in the study

**Patient group**	**Breast cancer and mutation status**	**Number of patients**	**Age in years, median (range)**	**Genetic predisposition**			**Parity**				**Hormone use at time of surgery**		
				**Family history**	***BRCA1***	***BRCA2***	**0**	**1 or 2**	**3 or 4**	**Data lacking**	**HRT**	**OC**	**Data lacking**
A	Cancer, non-*BRCA1/2*	21	50 (31–77)	4	NA	NA	8	4	9	0	3	1	0
B	Cancer, *BRCA1*	17	43 (23–81)	17	17	NA	4	10	0	3	4	1	3
C	Cancer, *BRCA2*	11	51 (33–74)	11	NA	11	0	7	3	1	0	1	2
D	Prophylactic mastectomy, *BRCA1*	12	37 (23–54)	12	12	NA	0	6	4	1	0	4	1
E	Prophylactic mastectomy, *BRCA2*	11	37 (31–51)	11	NA	11	0	5	5	1	0	0	1
F	Mammoplasty, non-cancer, non-*BRCA*	34	31 (20–68)	5	NA	NA	19	7	8	0	0	8	1

Patient groups included in the study and subsequently re-grouped according to the listed parameters for analysis of ALDH+ cells. The table presents selected clinical, genetic, and hormonal characteristics of patients.

NA, not applicable; HRT, hormone replacement therapy; OC, oral contraceptive.

Data on the following clinical parameters were available for the majority of the patients: indication for surgery, age at time of surgery, current or previous use and total duration of use of oral contraceptives and or hormone replacement therapy (HRT), age at menarche, number of live childbirths (parity), family history of breast cancer (1st or 2nd degree relative), and *BRCA1/2* mutation status. Table 1 presents information on the age, hormonal exposure, and reproductive status of the women included in the study.

Sixty-seven of the women were pre-menopausal, 35 were post-menopausal, and data were missing for four. The median age at onset of menopause was 49 years (range, 35–55 years). The numbers of live births among the women were as follows: para 0 in 32, para 1 in 11, para 2 in 28, para 3 in 25, and para 4 in four. The median age at menarche was 13 years (range, 10–18 years; data missing for 32 women). Data regarding exogenous hormone treatment status at the time of surgery were as follows: 70 women had no such treatment, 15 used oral contraceptives, seven received HRT, and six had a progestin intrauterine device. Sixty-five women had used oral contraceptives at sometime during their lives (median duration, 7.1 years), and 11 had received HRT at some point (median duration, 5.0 years). Clinical data and breast tissue samples representing 25 of the 28 patients in our previous study [15] were included in the present analysis.

### Histology and immunohistochemistry

If two tissue blocks from the same patient were of similar superior quality, both (from the same or both breasts) were analyzed in the same manner for quality assurance purposes. New sections were obtained from all relevant paraffin tissue blocks, and consecutive sections were H&E stained and used for immunohistochemistry, as described previously in detail [15]. Briefly, after antigen retrieval, the sections were incubated with mouse monoclonal antibodies directed against human ALDH1 A1 (aa 7–128, N-term, diluted 1:100; Becton, Dickinson and Company, Franklin Lakes, NJ, USA). Detection of immunoreactive sites was performed using a system based on horseradish peroxidase (HRP) and di-aminobenzidine (DAPI) labelling of primary antibodies raised in mice (Envision+ System-HRP, Dako, Glostrup, Denmark). Sequential incubations were performed with secondary antibodies and HRP-conjugated antibodies. Following the peroxidase reaction in DAPI and H_2_O_2_, the sections were rinsed and counterstained with hematoxylin, and then dehydrated and coverslipped in Mountex medium (Histolab Products AB, Göteborg, Sweden). To confirm the specificity of ALDH labelling, slides that were not incubated with the primary antibodies were included in each immunohistochemistry run.

The tissue sections selected for ALDH immunolabeling were assessed regarding the following features: tissue fragment size, epithelial atypia or hyperplasia, invasive carcinoma, and total number of TDLUs. The tissue areas available in each chosen block ranged in size from 48 to 621 mm^2^with a median value of 254 mm^2^. Areas displaying ductular or lobular hyperplasia, atypia, carcinoma *in situ* (four cases), or invasive carcinoma (10 cases) were excluded from further analysis. To evaluate the distribution and number of immunolabelled ALDH+ cells, 50 TDLUs were examined, guided by a grid, at evenly spaced intervals in each tissue section; if fewer than 50 TDLUs were present in a section, all the TDLUs were evaluated. The number of TDLUs found to be available for analysis of ALDH immunoreactivity in each patient ranged from 13 to 50 (mean, 45 TDLUs). Only ductules (i.e., no ducts or stroma) were evaluated. In our previous study [15], we described ALDH+ cells as being in a basal, luminal, or intermediate location. For each patient in the present study, the number of TDLU cross sections with ductular ALDH+ cells was recorded for each ductular level (luminal, intermediate, or basal) in relation to the total number of TDLU cross sections examined.

ALDH immunoreactive cells that were labelled either strongly or weakly were considered to be positive for ALDH. Furthermore, cells found in luminal and intermediate ductular locations were defined as non-basal (luminal plus intermediate) in some analyses, as indicated in the text. This was done because determination of cell location by ductular level is less accurate when ductules lack microscopically discernible lumina.

Blind testing of ALDH+ cell frequency in duplicate tissue samples from 13 patients yielded correlation coefficients of 0.45 (Pearson) and 0.53 (Spearman), indicating similarity between independent observations (r_s_ = 0.65, *p* = 0.01). These results confirmed the robustness of the analysis and demonstrated similarity between different areas of breast tissue in the same patient.

### Statistical analysis

The number of TDLUs with detectable ALDH+ cells and basal and non-basal localization of those cells were analyzed by linear regression using occurrence of ALDH+ cells in the mammoplasty group as the independent variable. Logistic regression was used to analyze differences between risk factor groups. Correlation between ALDH findings for duplicate samples from the same patient was analyzed using Spearman’s rank test and Pearson’s test. Two-tailed tests were performed to assess the level of statistical significance, and results with *p* < 0.05 were considered significant. SPSS 18 software (IBM, Armonk, NY, USA) was used for all statistical analyses.

### Ethics

The study was approved by the Research Ethics Committee for Southern Sweden (approval 11-92, 349-00).

## Results and discussion

### Division of patient inclusion groups A–F into analysis groups

Patients in inclusion groups A–F were divided into relevant groups on the basis of genetic and hormonal status to enable various analyses of ALDH+ cells.

### Distribution of ALDH+ cells

**Figure 1:**
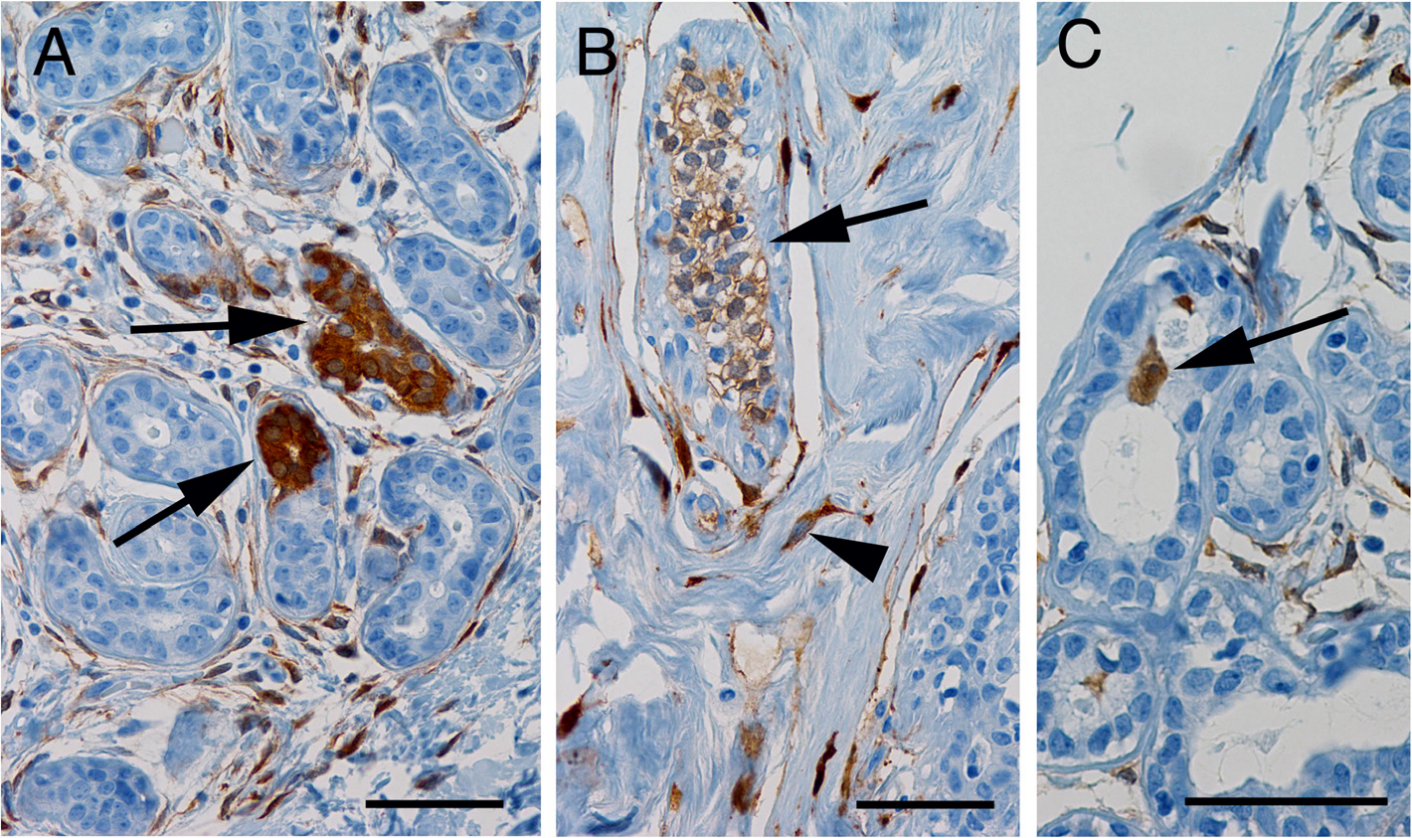
**Distribution of ALDH+ cells in benign breast ductules.** Brown staining indicates ALDH immunoreactivity. **A**, ALDH+ cell populations (arrows) located at luminal, basal, and intermediate ductular levels (levels described in detail elsewhere [15]). **B**, Micrograph showing an ALDH+ cell population (arrow) and ALDH+ stromal cells (arrowhead; as previously described [15]). **C**, Some ALDH+ cells are locatedin ductular bifurcations (arrow; cell type described previously [4,15]). All sections were counterstained with hematoxylin; scale bars in all three micrographs represent 50 μm.

ALDH+ cells were detected in TDLUs of 92 patients (87%), including the youngest (aged 20 years) and the oldest (aged 81 years). ALDH+ cells in ductules were morphologically similar to other ductular epithelial cells (Figure 1), and they were observed either as a few scattered cells or organized in groups comprising partial or entire ductular cross sections. The ALDH+ cells occurred primarily in the adluminal and intermediate levels (Figure 1B), and in some cases they were found in the adluminal level only (Figure 1C), or less often in all levels (Figure 1A). In several cases, ALDH+ cells extended across a ductular lumen, indicating the luminal aspect of a bifurcation. ALDH+ cells were located adluminally in 0–74% of TDLUs (median 6%), intermediately in 0–68% (median 6%), and basally in 0–30% (median 2%), thus implying predominantly non-basal localization of ALDH+ cells, as reported previously [4,15].

### ALDH+ cells in the six patient groups

ALDH+ cells were present in the epithelium at some ductular level in 90% of the subjects (19/21) in Group A (breast carcinoma with no *BRCA* mutations) and in a similar fraction (94%, 16/17) of the patients in Group B (breast carcinoma with *BRCA1* mutation), but in a smaller fraction (73%, 8/11) of those in Group C (breast carcinoma with *BRCA2* mutation). Also, ALDH+ ductular cells were detected in 100% (12/12) of the patients in group D (prophylactic mastectomy due to a *BRCA1* mutation) and 100% (11/11) of those in Group E (prophylactic mastectomy due to a *BRCA2* mutation). In Group F (mammoplasty patients with neither cancer nor *BRCA1/2* mutations), ALDH+ ductular cells were found in 76% (26/34) of the patients.

There was no statistically significant difference in the frequency of ALDH+ cells among the patient groups (*p* = 0.09), although a higher frequency was noted for patient Groups A–E combined compared to the mammoplasty group (*p* = 0.06, Fisher’s exact test). This finding, together with previous evidence that ALDH+ cells in different levels of the ductular epithelium are differentially associated with risk factors for breast cancer, [15] prompted a detailed analysis of patient subgroups regarding the occurrence of ALDH+ cells in the various levels of the ductular epithelium (see below).

### ALDH+ cells in patients with a family history of breast cancer but no BRCA1/2 mutations

**Figure 2:**
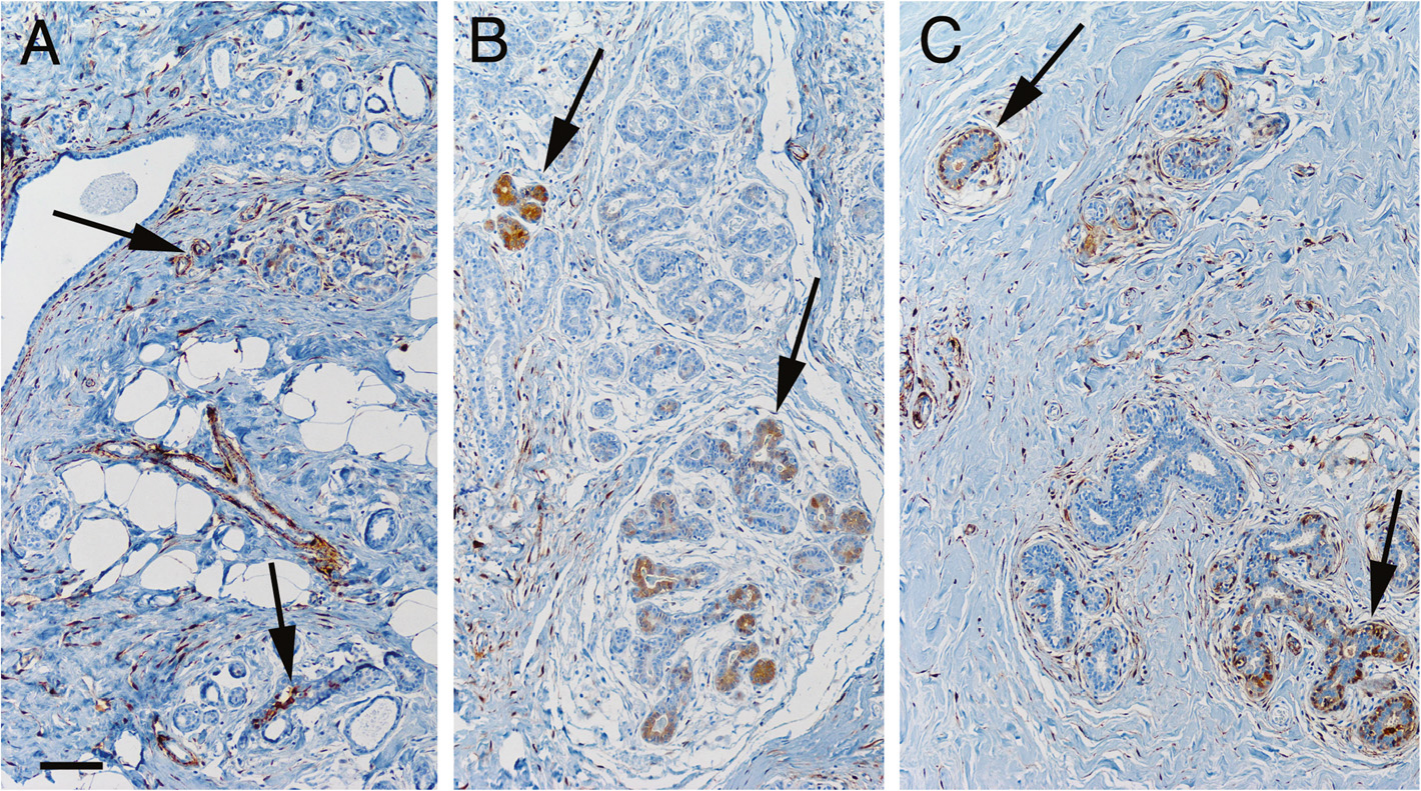
**Frequency of ALDH+ cells in ductules of benign breast tissue from pre-menopausal women with a family history of breast cancer.** ALDH+ cells were detected significantly more often in tissue from patients in this group than in control patients. **A**, Representative image of ALDH+ cell populations (arrows) in breast tissue from a woman with a family history of breast cancer but no *BRCA1/2* mutation. ALDH+ cells in the periphery of blood vessels, such as in the large Y-shaped structure in the center of this image, were detected in some cases (not analyzed further in this study). **B**, Image demonstrating ALDH+ cell populations (arrows) in benign breast tissue from a woman with a *BRCA1* mutation. **C**, Image showing ALDH+ cell populations (arrows) in benign breast tissue from a woman with a *BRCA2* mutation. Scale bar in A represents 100 μm in all three images.

Pre-menopausal patients with a family history of breast cancer but no *BRCA1/2* mutations were significantly associated with large numbers of ALDH+ cells at all ductular levels (*p* ≤ 0.01; Figure 2A). This association was independent of parity, age, and personal history of cancer, which indicates a positive relationship between ALDH expression and familial risk of breast cancer in women without *BRCA1/2* mutations. Notably, such an association with high frequency of ALDH+ cells was not observed at any ductular level in post-menopausal patients. Further information on these results is given in Table 2.

**Table 2:** Immunohistochemistry results

**Patients**	**Median percentage of TDLUs containing ALDH+ cells (range)**		
	**Luminal ductular level**	**Intermediate ductular level**	**Basal ductular level**
Pre-menopausal with family history of breast cancer, including patients with BRCA1 or BRCA2 mutation (n = 37)	12 (0–62)**	12 (0–53)**	2 (0–18)
Pre-menopausal with BRCA1 mutation (n = 18)	15 (2–50)*	15 (6–38)*	4 (0–18)
Pre-menopausal with BRCA2 mutation (n = 13)	14 (0–62)*	8 (2–53)*	0 (0–18)
Pre-menopausal without family history of breast cancer (n = 25)	6 (0–29)	6 (0–44)	0 (0–29)
Post-menopausal receiving HRT at the time of surgery (n = 7)	10 (0–38)*	8 (0–38)*	2 (0–21)
Post-menopausal not receiving HRT at the time of surgery (n = 24)	4 (0–32)	4 (0–34)	0 (0–18)
Nocancer, underwent mammoplasty (n = 34)	6 (0–32)	4 (0–44)	0 (0–29)

A statistical comparison of ALDH immunohistochemistry results for patients with and without a family history of breast cancer, and patients who were or were not receiving HRT at the time of surgery.

*P ≤ 0.05.

**P ≤ 0.01.

ALDH, aldehyde dehydrogenase 1 A1; TDLU, terminal duct lobular unit; HRT, hormone replacement therapy.

### ALDH+ cells in patients with a BRCA1 or BRCA2 mutation

Compared to breast tissue from mammoplasty patients, such tissue from pre-menopausal women who underwent preventive mastectomy due to *BRCA1* carrier status contained significantly larger numbers of ALDH+ cells at non-basal ductular levels (*p* = 0.03; see Figure 2B). A positive association of borderline significance (*p* = 0.06) was noted for ALDH+ cells located at the basal ductular level in the *BRCA1* mutation carriers. No difference in the occurrence or distribution of ALDH+ cells in breast tissue was observed between women with breast cancer and *BRCA1* gene mutations and those who underwent mammoplasty. Larger numbers of ALDH+ cells at non-basal ductular levels were discerned in tissue from pre-menopausal women with breast cancer and *BRCA2* gene mutations than in tissue from mammoplasty patients (*p* = 0.02; Figure 2C). The post-menopausal *BRCA1* and *BRCA2* patient subgroups showed no significant differences compared to the mammoplasty patients. Table 2 gives further information regarding these results.

It should also be mentioned that a higher frequency of ALDH+ cells in ductules was found in patients with *BRCA1* mutations than in those with *BRCA2* mutations. This may reflect the dissimilarities in oncogenesis between different types of *BRCA* mutations that have been described by other investigators [16].

### ALDH+ cells in relation to age and menarche

**Figure 3:**
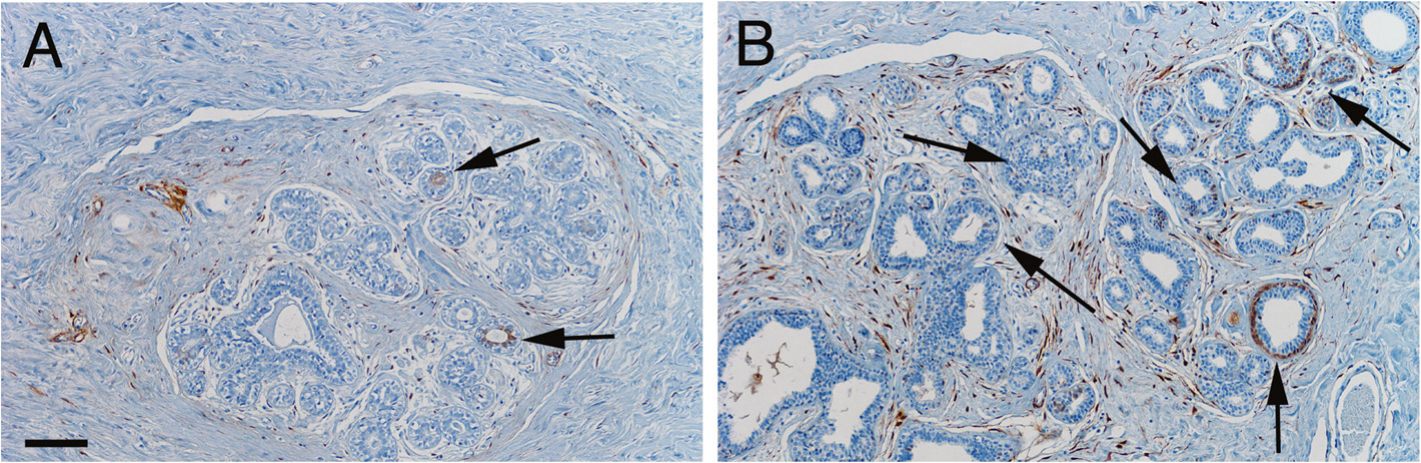
**Frequency of ALDH+ cells in benign ductules of pre-menopausal women of different ages.** Representative images of benign ductules in breast tissue from a 31-year-old patient **(A)** and from a and a 50-year-old patient **(B)** demonstrating significantly higher frequency of ALDH+ cells (arrows) with increasing age. Scale bar in A represents 100 μm in both images.

Among pre-menopausal patients without *BRCA* mutations, age was positively associated with the frequency of ALDH+ cells at all ductular levels (*p* ≤ 0.01; Figure 3), even after adjusting for parity and family history of breast cancer (*p* = 0.01), which suggests that risk factors have a stronger influence with increasing age. This association was not found within the post-menopausal group. Age at menarche was not correlated with the frequency or the distribution of ALDH+ cells.

### ALDH+ cells in relation to parity

**Figure 4:**
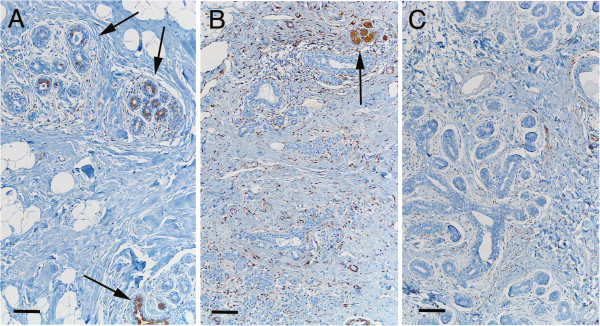
**Frequency of ALDH+ cells in benign ductules of pre-menopausal women with different parities.** Representative images of benign ductules in pre-menopausal patients demonstrate a significant decrease in frequency of ALDH+ cells (brown) with increasing parity in this patient subgroup. **A**, Image illustrating the relatively large number of ductular ALDH+ cells (arrows) in breast tissue from a 39-year-oldnulliparous woman. **B**, Image of breast tissue from a 38-year-old two-parous woman showing therelatively small number of TDLUs containing ductular ALDH+ cells (arrow), and scattered ALDH+ cells in the stroma. **C**, Image revealing the lack of ductular ALDH+ cells in breast tissue from a 41-year-old four-parous woman. Scale bars: 100 μm.

In breast tissue from pre-menopausal patients with or without cancer or *BRCA* mutations, adjusted for age, the frequency of ALDH+ cells at all three ductular levels was significantly lower with increased parity (*p* ≤ 0.03; see Figure 4). This relationship was not observed in the post-menopausal group. Thus the lower risk of breast cancer associated with higher parity [17] may be related to the lower frequency of ALDH+ cells in high-parity patients. However, this possibility needs to be further investigated using a larger data set and focusing on mutation status.

### ALDH+ cells in relation to HRT and oral contraceptives

**Figure 5:**
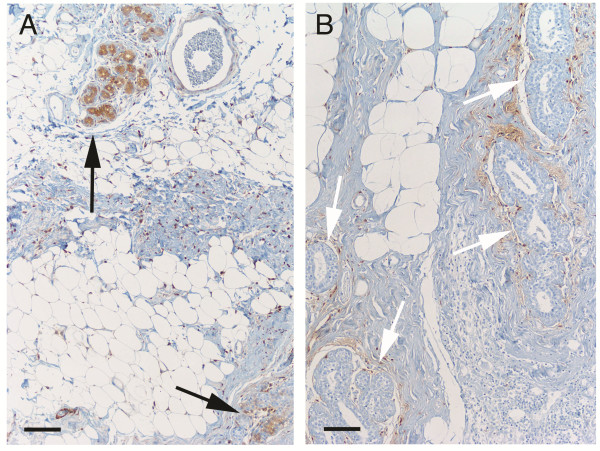
**Frequency of ALDH+ cells in benign ductules of post-menopausal women with and without HRT at the time of surgery.** Representative images of tissue from post-menopausal women are presented that demonstrate the significantly increased frequency of ALDH+ cells in breast tissue from women receiving HRT treatment at the time of surgery. **A**, Image illustrating the relatively high number of TDLUs containing ALDH+ cells (arrows) in benign breast tissue from a 70-year-old woman receiving HRT at the time of cancer surgery. **B**, Image of benign breast tissue from a 74-year-old woman who was not receiving HRT at the time of surgery, showing the presence of relatively few or complete absence of ALDH+ cells (white arrows indicate ductules with no ALDH+ cells). The benign tissue from this woman also contained ALDH+ stromal cells, as demonstrated in all patient groups. Scale bars: 100 μm.

No correlation was found between the use of oral contraceptives at the time of surgery and the frequency of ALDH+ cells at any ductular level in breast tissue, indicating that the hormonal impact of oral contraceptives alone is not sufficient to override physiological hormonal changes. However, compared to post-menopausal women who were not using HRT at the time of surgery, those who were on such therapy had larger numbers of ALDH+ cells in tissue at basal (p = 0.025) and non-basal (*p* = 0.03) levels (Figure 5 and Table 2), and this difference was independent of other risk factors. In the post-menopausal group, HRT was the only hormone intake parameter that showed a statistically significant correlation with a high frequency of ALDH+ cells, which agrees with evidence that HRT postpones TDLU involution and increases the risk of cancer [18-21]. This finding also suggests that ongoing HRT may be one of the factors that influence the number of ALDH+ cells.

In contrast, neither the total duration of contraceptive pill usage nor the duration of HRT was associated with the frequency of ALDH+ cells, which suggests that exogenous hormones have only a transient effect on the occurrence of ALDH+ cells in benign breast tissue, and is consistent with previously reported data on sex hormone therapies suggesting that ongoing or recent intake of HRT increases the risk of breast cancer [19].

### ALDH+ cells in relation to cancer versus non-cancer

The present findings support the hypothesis that ALDH+ breast cell populations participate in oncogenesis. We found several associations between high frequencies of ALDH+ cells in benign breast tissue and clinical risk factors for cancer. Also, statistical analysis of subgroups demonstrated significant correlations between ALDH+ cells and family history of breast cancer, parity, and HRT exposure (see Table 2). Larger studies are needed to assess other risk factors for breast cancer in this context.

### Methodological considerations

The patients evaluated in the present study were treated between 1984 and 2010, and the surgical techniques and oncological treatment protocols being applied during that period were subject to changes. However, the potential impact of those modifications on the results of our investigation can be assumed to be negligible for the following reasons: (1) patients who received neoadjuvant therapy were excluded; (2) possible influence of variation in antigenicity related to heterogeneous tissue fixation was reduced to a minimum by using ALDH positivity in stromal cells as internal control, and by considering all cells exhibiting weak or strong immunoreactivity as positive for expression of ALDH.

Our study demonstrated associations between ALDH+ cell frequencies in benign breast tissue and risk factors for breast cancer, but we found no significant relationship between ALDH+ cell frequencies in benign tissue and the co-presence of cancer. This finding might be explained by the influence of temporal and spatial factors of a developing cancer; more specifically, that the cancer is established after the occurrence of a risk factor, and the tumor can overgrow ALDH+ cells.

In our previous study conducted using the same methods [15], a higher frequency of ALDH+ ductular cells was found in mammoplasty patients than in patients with established breast cancer. In contrast, in the present investigation, data on mammoplasty patients were used to allow comparison of non-breast cancer and breast cancer patients, the latter group including many individuals with a familial risk of breast cancer. However, inasmuch as increased cell mass in a breast predisposes to cancer [22,23], it would be most suitable to obtain control tissue from patients without breast disease, not from those with breast hyperplasia.

## Conclusion

The present study demonstrated a positive association between the frequency of ALDH+ ductular cells in benign female breast tissue and several well-known risk factors for breast cancer. These findings suggest that ALDH cell positivity in microscopically normal breast tissue should be further evaluated as a potential marker for the risk of breast cancer, especially in patients with familial breast cancer with or without BRCA1/2 mutations.

The largest differences in ALDH+ cell frequency between risk groups were observed in the luminal and intermediate (non-basal) ductular locations. Our data indicate that ALDH-expressing cells may play a yet undefined role in the development of female breast cancer, and they also suggest that immunohistochemical analysis of ALDH can aid prediction of the risk of breast cancer. It is conceivable that having a high number of ALDH+ stem cells promotes a high number of progenitor cells, and that the latter are susceptible to oncogenesis. The current results underline the need for further research to assess the previously proposed stem and/or progenitor qualities of ALDH+ cells in normal breast tissue and their potential role in carcinogenesis.

## Competing interests

The authors declare that they have no competing interests.

## Authors’ contributions

BLI conceived this study, coordinated the laboratory work, examined the microscopic material, participated in the data analysis, and coordinated the writing of the manuscript. BH participated in the experimental design and in the writing of the manuscript. HJ provided clinical data and helped write the manuscript. PA helped design the study and helped write the manuscript. HO helped design the study, was responsible for ethical approval, provided clinical data, performed data analysis, and helped write the manuscript. All authors read and approved the final manuscript.

## Pre-publication history

The pre-publication history for this paper can be accessed here:

http://www.biomedcentral.com/1472-6890/13/28/prepub
